# Changes in mental illness stigma over 30 years – Improvement, persistence, or deterioration?

**DOI:** 10.1192/j.eurpsy.2022.2337

**Published:** 2022-11-04

**Authors:** Georg Schomerus, Stephanie Schindler, Christian Sander, Eva Baumann, Matthias C. Angermeyer

**Affiliations:** 1 Department of Psychiatry, University of Leipzig Medical Center, Leipzig, Germany; 2 Department of Journalism and Communication Research, Hannover University of Music, Drama, and Media, Hannover, Germany; 3 Center for Public Mental Health, Gösing am Wagram, Austria

**Keywords:** Representative population study, social distance, stigma, time-trend

## Abstract

**Background:**

Large efforts have been made to erase the stigma of mental illness, but it is unclear whether they have succeeded on a population level. We examine how attitudes toward people with depression or schizophrenia have evolved in Germany since 1990, and whether there are different developments for both disorders.

**Methods:**

Using data from the three decades, four wave repeated cross-sectional representative population study in the “old” (western) states in Germany with surveys in 1990 (*n* = 2,044), 2001 (*n* = 4,005), 2011 (*n* = 1,984), and 2020 (*n* = 2,449), we calculate time-trends for social distance and emotional reactions toward someone with major depression or acute schizophrenia.

**Results:**

Social distance worsened in six out of seven situations for schizophrenia, whereas improving in two out of seven situations for depression. Emotions related to fear and uneasiness increased for schizophrenia, whereas tending to decrease for depression. Pro-social reactions like the desire to help increased for depression, but decreased for schizophrenia. Initially observed differences, favoring depression over schizophrenia, widened over the 30-year study period. For schizophrenia, the biggest negative changes occurred between 1990 and 2001, whereas some improvements with regard to depression occurred more recently.

**Conclusion:**

Contrary to expectations, stigma has become more severe regarding acute schizophrenia in Germany over the last 30 years, whereas only slightly improving for depression. The apparent normalization of mental health problems seems not to directly translate into improving attitudes toward people with severe mental illness. Re-focusing of anti-stigma efforts on people with severe mental illness seems necessary.

## Introduction

Over the last several decades, a strong concerted effort has been mounted to improve the attitudinal context in which mental illness is experienced. Scores of studies evaluating strategies designed to improve public attitudes have been undertaken and several meta-analyses of these studies have been generated [1, 2], indicating that multiple approaches can be effective in improving public knowledge, attitudes, and beliefs about mental illnesses. Numerous anti-stigma and awareness initiatives have worked in many countries on a national or regional level toward overcoming the taboo surrounding mental illness [3]. In Germany, different to other European countries such as England [4], Sweden [5], Denmark [6], or the Czech Republic [7], no national anti-stigma initiative has been launched, but a multitude of local and regional initiatives have aimed at improving mental health literacy and reducing stigma. Among them, the most prominent were the Word Psychiatric Association’s campaign “Open the doors” [8], the “German Alliance Against Depression” [9], “Irrsinnig Menschlich” [10], and “psychenet,” the Hamburg Network for Mental Health [11]. All of these campaigns started in the early 2000s. In 2009, the professional organization of German psychiatrists (DGPPN) established a national anti-stigma award. Alongside these efforts and the dramatic changes in the care and treatment of people with mental illnesses following psychiatric reform [12], there is a general notion that more people come out with their personal mental health struggles [13], and that conversations about mental health issues have become easier [14], [15]. Trends like the increasing demand for psychotherapy in Germany have been explained by “the overall declining stigmatization of mental illness” [16]. So the question arises whether these accumulated efforts finally have population level impact on attitudes toward people with mental illness. Do we finally see the “end of the story” about mental illness stigma [17]?

In contrast to these optimistic expectations, trend studies so far have not shown a general improvement of stigma. With regard to schizophrenia, a meta-analysis of population studies showed worsening stigma between 1990 and 2006 [18]. Developments for depression seem more promising, with unchanging [18] and recently, in a trend-study from the USA, even improving attitudes [19].

In this study, we present evidence from the three decades (1990–2020), four wave repeated multiple cross-sectional studies in Germany that provide population-level evidence about changes in public attitudes concerning major depression and schizophrenia. Because our study is the longest running population-based study that has used identical measurement consistently across waves, it provides a unique opportunity to evaluate population-level changes that may or may not have followed the strong efforts to improve attitudes toward mental illnesses. Using un-labeled case vignettes of a person with either schizophrenia or depression in each survey, we test (a) whether attitudes toward individuals with mental illness have improved over the last 30 years, and (b) whether attitudes have developed differently for either depression or schizophrenia. These hypotheses will be examined by comparing attitudes at the baseline (1990) and last follow-up (2020). Using data from all four surveys 1990, 2001, 2011, and 1990, we further explore (c) whether any observable changes were particularly pronounced in any of the three decades covered by our study.

## Methods

Our study is based on four population surveys conducted among people aged 18 years and older, living in the “old” West German federal states. We excluded respondents in the “East,” the former German Democratic Republic, since our baseline survey in 1990 was conducted pre-re-unification and did not cover East Germany, and any comparative analyses between surveys need to be based on the same region. Surveys were conducted face-to-face in 1990 (*n* = 2,044, response rate 70%), 2001 (*n* = 4,005, response rate 65%), 2011 (*n* = 1,984, response rate 64%), and 2020 (*n* = 2449, response rate 57%). In all four surveys, samples were drawn using an identical random sampling procedure with three stages (for details, see the Supplementary Materials). Supplementary Table S1 shows the sociodemographic characteristics (gender, age, and education) that were collected in all surveys, and the according numbers of the general population at that time. Except for education, where highly educated people were under-represented in 2011 and 2020, our samples can be considered representative of the German population. The study was approved by the review board of Greifswald University Medical Center (BB 195/18).

### Interview

All surveys were carried out as in-person, face-to-face interviews by trained interviewers using paper and pencil. All participants gave verbal informed consent. The fully structured interviews were identical regarding wording and the sequence of questions. In 2020, due to the COVID-19 pandemic, respondents were offered the opportunity to complete the interview by themselves, while the interviewer was waiting outside. This procedure was chosen by 18.6% of respondents (*n* = 456), all other interviews were conducted face-to-face as in previous surveys. Interviews started by presenting a diagnostically unlabeled psychiatric case history (vignette). Respondents were randomly assigned either a description of someone with schizophrenia or major depressive disorder. The symptoms described fulfilled the criteria of the Diagnostic and Statistical Manual of Mental Disorders(1987 revision, III-R) for the respective disorder. The wording of both vignettes is provided in the Supplementary Material. Before being used in the first survey, each vignette had been rated by five experts in psychopathology, confirming the correct diagnosis. The gender of the vignette varied at random in 1990, 2011, and 2020. In 2001, only male characters were provided. The depression vignette was presented to *n* = 991 respondents in 1990; *n* = 2,018 in 2001; *n* = 985 in 2011; and *n* = 1,231 in 2020. The schizophrenia vignette was presented to *n* = 1,053 respondents in 1990; *n* = 1,987 in 2001; *n* = 999 in 2011; and *n* = 1,218 in 2020.

### Attitudes toward people with mental disorders

We measured two core components of stigma as conceptualized by Link et al. [20]: negative emotional reactions and the desire for social distance. We used the Social Distance Scale (SDS) developed by Link et al. to assess the respondents’ willingness to accept the person described in the vignette in hypothetical situations like renting a room, working together, or having as a neighbor [21]. Answers were given on five-point Likert scales with the anchors “very likely” (1) and “very unlikely” (5). Higher scores thus indicate a stronger desire for social distance.

To assess emotional reactions, we used the Emotional Reactions toward Mental Illness Scale (ERMIS) [22], which contains 10 items, representing “pro-social feelings” (e.g., I feel the need to help him/her); “fear” (e.g., He/she scares me); and “anger” (e.g., I feel annoyed by him/her). Each item was answered on a five-point Likert scale with the anchors “agree completely” (1) and “disagree agree completely” (5). To enable a more intuitive interpretation, we reversed the scores so that higher values indicate stronger emotional reactions. Both SDS and ERMIS were analyzed on an item level to capture changes with regard to different social situations (SDS) or emotional reactions (ERMIS).

### Statistical analysis

Statistical analyses are described in detail in the Supplementary Material. In brief, we performed multiple regression analyses of the individual five-point items representing emotional reactions to, and the desire for social distance from people with schizophrenia and depression as criteria using an ordinary least squares estimator. Survey, vignette (depression or schizophrenia), and interaction of survey*vignette entered the analyses as predictors. Gender of the vignette, age of respondents, and gender of respondents, respectively, entered the analyses as covariates. Respondents of “diverse” gender were excluded from the analyses due to low occurrence. While question 1 describes the *30-year trends* per vignette, question 2 examines whether these trends followed different trajectories for both disorders as indicated by the interaction term time*vignette. The interaction term indicates whether the *difference* in attitudes between both disorders increases over time. Question 3, finally, looks in more detail at the *time course of attitudes* related to each vignette. For more details, see the expanded methods section in the Supplementary Material. To rule out potential confounding effects of self-administered interviews in 2020, we conducted sensitivity analyses of all major results excluding these cases. All *p-*values were corrected for separate testing of items and vignettes using the Benjamini–Hochberg procedure to control the false discovery rate at *q* = 0.05.

## Results

### 1. Have attitudes toward someone with mental illness improved over the last 30 years?

**Figure 1.**
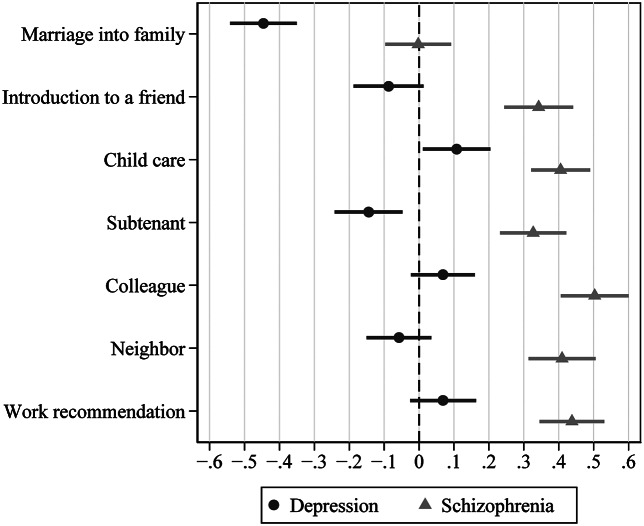
Changes in the desire for social distance from someone with schizophrenia or depression 1990–2020. Selected contrasts of multiple regression analyses with items of the Social Distance Scale as criterion and period, vignette, interaction of period*vignette, age, and gender of vignette or respondents as predictors. The contrast estimates describe the change from 1990 to 2020 per vignette, bars indicate 95% confidence intervals. Higher values indicate an increasing desire for social distance.

Figure 1 displays the results of regression analyses comparing levels of *social distance* in 2020 compared to 1990, depicting the predicted changes of the mean value on the five-point Likert scale, controlled for age and gender of the respondents, and gender of the vignette (see the Supplementary Table S2 for the model fit statistics of each item). Social distance toward someone with *schizophrenia* increased in all situations by 0.33 to 0.50 points, except for “marriage into ones family,” where it did not change. Social distance toward someone with *depression*, in turn, improved in two of seven situations, marrying into one’s family (−0.45), and letting a room (−0.14), and increased in one instance, taking care of a child (0.11), whereas it did not change significantly in the other four situations. Sensitivity analyses (excluding the 18.6% of respondents in 2020 who answered the questionnaire in writing) yielded similar results, with a numerically slightly stronger desire for social distance in 2020 (Supplementary Table S3).

**Figure 2.**
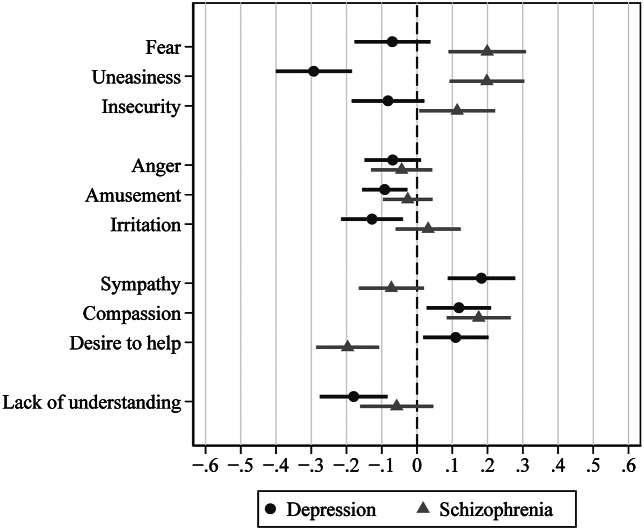
Changes in emotional reactions toward with schizophrenia or depression 1990–2020. Selected contrasts of multiple regression analyses with items of the Emotional Reaction toward Mental Illness Scale as criteria and period, vignette, interaction of period*vignette, age, and gender of vignette or respondents as predictors. The contrast estimates describe the change from 1990 to 2020 per vignette, bars indicate 95% confidence intervals. Higher values indicate an increase in emotional reactions.

Figure 2 shows how *emotional reactions* have changed in 2020 compared to 1990. Supplementary Table S4 lists the model fit statistics for each emotional reaction. Regarding *schizophrenia*, both fear and feeling uneasy increased significantly by 0.20 points, whereas the desire to help decreased by −0.20 points. The increase in insecurity (*b* = 0.11; 95% CI 0.01–0.22) was below significance (*p* = 0.063) after Benjamini–Hochberg correction for false discovery rates. Regarding *depression*, feeling uneasy (−0.29) and irritation (−0.13) decreased, whereas the desire to help and feeling sympathy increased by 0.11 and 0.18 points, respectively. Compassion increased in both disorders, amusement, and lack of understanding decreased significantly in depression, but since survey*vignette interactions were insignificant (see Supplementary Table S4), this could also be parallel developments for both disorders being only more pronounced in depression. Sensitivity analyses excluding respondents who chose to answer the questionnaire in writing in 2020 yielded generally similar results, with some minor changes in effect sizes and significance (e.g., the increase in insecurity regarding schizophrenia being now significant, whereas the decrease in irritation regarding depression being no longer significant; Supplementary Table S5).

Overall, we thus observed worsening attitudes toward a person with schizophrenia, and some improvement of attitudes toward a person with depression. Only compassion increased significantly for both disorders.

### 2. Have attitudes developed differently for either depression or schizophrenia?

Table 1 shows the results of the regression analyses focusing on the difference in the *desire for social distance* between both disorders, and whether this difference increased over time. It confirms that with all items, the difference in social distance between both vignettes has increased since 1990. For example, the initial difference in willingness to introduce the person to a friend was 0.13 in 1990. This difference further increased by 0.43 from 1990 to 2020. Rejecting someone as a neighbor differed by 0.16 points between both disorders in 1990, a difference further increasing by 0.47 in 2020. Sensitivity analyses excluding self-administered interviews in 2020 yielded similar results (Supplementary Table S6).

**Table 1.** Differences in the desire for social distance from someone with schizophrenia or depression, 1990–2020.

		Estimates			Test statistics	
Desire for social distance related to…	Parameter	*b*	SE (robust)	[95% CI]	*t*	*p*
Marriage into family	Vignette (1990)	0.11	0.05	0.01, 0.20	2.12	0.034*
	2020*vignette	0.44	0.07	0.31, 0.58	6.48	<0.001***
Introduction to friend	Vignette (1990)	0.13	0.05	0.02, 0.23	2.39	0.020*
	2020*vignette	0.43	0.07	0.29, 0.57	5.99	<0.001***
Child care	Vignette (1990)	0.27	0.05	0.17, 0.37	5.31	<0.001***
	2020*vignette	0.30	0.07	0.17, 0.43	4.55	<0.001***
Subtenant	Vignette (1990)	0.22	0.05	0.12, 0.32	4.40	<0.001***
	2020*vignette	0.47	0.07	0.34, 0.61	6.81	<0.001***
Colleague	Vignette (1990)	0.13	0.05	0.03, 0.22	2.49	0.020*
	2020*vignette	0.43	0.07	0.30, 0.57	6.39	<0.001***
Neighbor	Vignette (1990)	0.16	0.05	0.06, 0.26	3.21	0.003**
	2020*vignette	0.47	0.07	0.33, 0.60	6.86	<0.001***
Work recommendation	Vignette (1990)	0.12	0.05	0.02, 0.22	2.39	0.020*
	2020*vignette	0.37	0.07	0.24, 0.50	5.46	<0.001***

*Note*: Results of multiple regression analyses of the items of the Social Distance Scale, with period, vignette, interaction of period*vignette, age, and gender of vignette or respondents as predictors (*n* = 10,361–10,380). We show selected estimates (the initial gap between schizophrenia and depression in 1990, and the increase of this gap until 2020) with *p-*values corrected for separate testing of items using the Benjamini–Hochberg procedure to control the false discovery rate at *q* = 0.05. *p < 0.05, **p < 0.01, ***p < 0.001.

*Abbreviations*: CI, confidence interval; SE, standard error.

Table 2 shows the results of the regression analyses focusing on the differences in emotional reactions between both disorders. Feelings of fear, uneasiness, or irritation were all bigger for schizophrenia compared to depression in 1990, and this gap widened until 2020. For example, schizophrenia evoked 0.25 points more fear than depression in 1990, and this difference increased further by 0.27 points until 2020. Anger and lack of understanding were also greater for schizophrenia in 1990, but this difference did not increase further.

**Table 2.** Differences in emotional reactions to someone with schizophrenia or depression 1990–2020.

		Estimates			Test statistics	
Emotional reaction	Parameter	*b*	SE (robust)	[95% CI]	*t*	*p*
Fear	Vignette (1990)	0.25	0.06	0.14, 0.36	4.40	<0.001***
	2020*vignette	0.27	0.08	0.12, 0.42	3.43	0.002**
Uneasiness	Vignette (1990)	0.15	0.06	0.04, 0.27	2.74	0.010*
	2020*vignette	0.49	0.08	0.34, 0.64	6.37	<0.001***
Insecurity	Vignette (1990)	0.23	0.06	0.12, 0.34	4.16	<0.001***
	2020*vignette	0.20	0.08	0.05, 0.34	2.59	0.019*
Anger	Vignette (1990)	0.16	0.04	0.07, 0.24	3.56	<0.001***
	2020*vignette	0.03	0.06	−0.09, 0.14	0.42	0.675
Amusement	Vignette (1990)	0.06	0.04	−0.01, 0.14	1.75	0.089
	2020*vignette	0.07	0.05	−0.03, 0.16	1.34	0.224
Irritation	Vignette (1990)	0.16	0.05	0.07, 0.25	3.35	0.002**
	2020*vignette	0.16	0.06	0.03, 0.29	2.46	0.023*
Sympathy	Vignette (1990)	−0.11	0.05	−0.21, −0.01	−2.26	0.030*
	2020*vignette	−0.26	0.07	−0.39, −0.12	−3.78	<0.001***
Compassion	Vignette (1990)	−0.06	0.05	−0.15, 0.04	−1.17	0.244
	2020*vignette	0.06	0.07	−0.07, 0.18	0.86	0.433
Desire to help	Vignette (1990)	0.11	0.05	0.02, 0.21	2.36	0.026*
	2020*vignette	−0.31	0.07	−0.44, −0.18	−4.68	<0.001***
Lack of understanding	Vignette (1990)	0.25	0.05	0.14, 0.36	4.64	<0.001***
	2020*vignette	0.12	0.07	−0.02, 0.26	1.70	0.128

*Note*: Results of multiple regression analyses of the items of the emotional reactions toward Mental Illness Scale, with period, vignette, interaction of period*vignette, age, and gender of vignette or respondents as predictors (*n* = 10,367–10,383). We show selected estimates (the initial gap between schizophrenia and depression in 1990, and the increase of this gap until 2020) with *p*-values corrected for separate testing of items using the Benjamini–Hochberg procedure to control the false discovery rate at *q* = 0.05. *p < 0.05, **p < 0.01, ***p < 0.001.

*Abbreviations*: CI, confidence interval; SE, standard error.

Pro-social reactions like sympathy were generally lower for schizophrenia than for depression. Feelings of sympathy, for example, were lower for schizophrenia in 1990 by −0.11 points, a difference that further increased until 2020 by −0.26 points. The desire to help, starting from a difference of 0.11 points in favor of schizophrenia, declined in schizophrenia significantly by −0.31 points in 2020, thus turning in favor of depression. Only amusement and compassion were similar between both disorders, and no significant difference emerged over 30 years.

Overall, the difference in attitudes between both disorders increased considerably over the last 30 years, always to the disadvantage of someone with schizophrenia. These results were confirmed in sensitivity analyses excluding those who answered the questionnaire in writing in 2020 (Supplementary Table S7).

### 3. Have there been times when any change in attitudes was particularly pronounced?

To answer our third research question, we looked at the magnitude and statistical significance of any changes within each decade (Supplementary Tables S8 and S9). In schizophrenia, the biggest and most statistically significant changes occurred between 1990 and 2001. For example, reluctance to have this person as a co-worker increased by 0.47 points in the earliest decade, with insignificant subsequent changes. Fear increased by 0.15 between 1990 and 2001, whereas later changes were smaller and statistically not significant. Similarly, uneasiness increased by 0.26 between 1990 and 2001, with insignificant later changes. Only a few significant changes occurred in the later decades. Sympathy, for example, decreased by −0.11 between 2011 and 2020.

With depression, attitude changes are seen in the more recent decades. For example, unwillingness to sublet a room to someone with depression decreased by −0.17 between 2001 and 2011, as did unwillingness to have as a neighbor (−0.16). Uneasiness decreased by −0.17 between 2001 and 2011. Between 2011 and 2020, anger decreased by −0.14, and lack of understanding by −0.19 (Supplementary Table S9). Overall, the increase in stigma toward someone with schizophrenia seems to have been strongest in the course of the 1990s. Some of the improvements observed for depression have occurred in later decades. Sensitivity analyses excluding those who answered in writing in 2020 broadly confirmed these results (Supplementary Tables S8 and S9).

## Discussion

Summarizing our findings, we found that people’s attitude toward schizophrenia are more negative than toward depression. Moreover, attitudes toward someone with schizophrenia have generally changed to the worse over the last 30 years. For depression, attitudes improved with regard to single items, particularly with regard to emotional reactions, where people increasingly felt less uneasy and more sympathetic. The biggest negative changes regarding schizophrenia occurred between 1990 and 2001, whereas some improvements occurred for depression recently. The initially observed divide in attitudes toward both disorders thus widened over the entire period of 30 years.

Before discussing potential reasons for these developments, we have to consider the strengths and weaknesses of our study. Spanning 30 years, our study is the longest vignette-based time-trend study on the stigma of mental illness so far. Being based on four cross-sectional surveys with sample sizes of *n* = 985 to *n* = 2,018 respondents per vignette, we were able to perform complex statistical modeling of time trends and control our results for multiple testing of hypotheses. Case vignettes show trends in reactions to identical, specific situations, which is a major strength of this study, but also comes at a cost: our study based on two descriptions of acute episodes of major depression or schizophrenia does not allow conclusions on attitudes toward people who have, for instance, recovered from their mental illness, or who are currently treated for a mental health problem. Another weakness of our study is the declining response rate (from 70% in 1990 to 57% in 2020). Declining response rates are a common phenomenon in attitude research, although face-to-face studies like the present study still yield higher response rates than telephone surveys [23]. Due to the COVID-19 pandemic, 18.6% of respondents in 2020 opted to answer the questionnaire in writing. By conducting sensitivity analyses excluding these respondents, yielding generally similar results, however, we could show that these circumstances did not impair the validity of our findings.

Results of our study are in stark contrast to studies showing improving attitudes toward people with unspecified “mental illness” or “mental health problems.” In England, for example, more than 10 years of the highly visible Time to Change initiative from 2008 to 2021 have been accompanied by a constant improvement of attitudes toward people with mental health problems, starting about 3 years after the launch of the campaign [24]. Similarly, in Germany, the perceived stigma of a “former mental patient” improved considerably between 1990 and 2011 [25]. For instance, while 30% of the population agreed in 1990 that “most people would willingly accept a former mental patient as a close friend,” this percentage had risen to 43% in 2011.

So, our findings provoke two questions which we will discuss in the following: First, why have attitudes toward “someone with mental illness” develop more favorably compared to attitudes toward someone who is actually described as having a mental illness? Second, why have attitudes toward someone with schizophrenia and depression evolved differently since 1990?

### Normalization of mental health problems

It has been argued that a more open discourse on mental health problems contributes to a normalization and broadening of the significance of terms like “mental health problems” [26]. In fact, in England, the public conception of what constitutes a mental illness has expanded during the Time to Change campaign. While in 2009, 58% stated that “stress” is a type of mental illness, this percentage rose to 66% in 2017. Similarly, grief was considered a mental illness by 49% in 2009 and by 57% in 2017 [24]. If the public conception of “mentaI illness” is broadening, people might associate more common, less severe mental health problems with labels that are not backed up by detailed descriptions of symptoms or impairments. The observed improvement in attitudes might then at least in part be due to these less severe images that come to people’s minds when answering respective survey questions. Our study suggests that this normalization of mental health problems, in general, does not easily translate into more tolerant attitudes toward someone with a defined, acute, severe mental health problem as described by the respondents in our study.

### A growing gap

A growing divide between attitudes toward someone with depression and schizophrenia has also been observed in the USA [19], where the desire for social distance toward someone with depression declined between 2006 and 2018, a trend not seen in alcohol dependence or schizophrenia. Depression has received considerable public attention in recent years, for example, through the work of the European Alliance against depression [27]. Furthermore, the disclosure of major depression among celebrities and the resulting media and public awareness of the issue may have lowered fears of contact and increased the perceived proximity to people affected [28]. The rising popularity of this diagnosis has even become the subject of historical analyses [29]. Increasingly, concepts like “burnout” are being used to describe states of depression [30–32], linking depression to the stresses of everyday life in modern society. A trend study of continuum beliefs in Germany found the percentage of people agreeing with continuum beliefs for depression increased from 43 to 46% during the period of 2011 to 2020 [33]. During that time, rejection of the notion that the symptoms of depression are incomprehensible or unfamiliar rose by 9 and 10%, to 57 and 54%, respectively. Continuum beliefs with regard to schizophrenia, in contrast, decreased from 26 to 20% [33]. People thus seem to be ever more ready to “take the role” of someone with symptoms of depression. In his article “A symbolic interactionist view of psychosis,” Rosenberg (1984) argues that being able to take the role or the perspective of someone in trouble is signifying the “sanity” of a certain state of mind [34], in contrast to the “insanity” of states with incomprehensible behavior. Arguably, depression is increasingly considered a “sane” reaction to a stressful environment, contrasting with a growing notion of schizophrenia as being unrelatable and “insane.” In our data, this is mirrored in the growing sympathy for someone with depression, the growing readiness to help someone with depression, and the declining readiness to help someone with schizophrenia (Figure 2).

Experimental and correlational studies have shown that biological etiological beliefs about mental disorders increase stigma, by increasing notions of dangerousness and differentness [35–38]. In fact, the strongest increase in schizophrenia stigma in our study occurred during the so-called “Decade of the brain” in the 1990s, which was accompanied by increasing beliefs in biological causes for both depression and schizophrenia [18]. If biogenetic illness, beliefs are among the drivers of the increase in schizophrenia stigma, our results suggest an illness-specific effect, probably by reinforcing the high (and rising) levels of perceived differentness and incomprehensibility found particularly with regard to schizophrenia [33, 38].

Other findings corroborate the notion of a growing gap between attitudes related to both disorders. Media reports on depression are frequent, and are often concerned with treatment modalities, whereas the reports on schizophrenia are most often concerned with violence and crime [39]. While notions of dangerousness of someone with schizophrenia are thus reinforced, potentially contributing to the increasing stigma surrounding schizophrenia [19, 40], effects of media reporting regarding depression could be beneficial: They seem to have translated, for example, into a growing recognition of the healthcare needs associated with depression. A trend study of funding preferences in healthcare among the general public revealed that funding for the care of people with depression has become increasingly popular over the last 20 years, from ranking second-last out of nine medical and mental disorder in 2001 to ranking fourth in 2020 (after cancer, cardiovascular diseases, and diabetes, but ahead of Alzheimer’s, Rheumatism, and AIDS) [41]. At the same time, funding for people with schizophrenia in 2020 ranked second-last [41].

In conclusion, the stigma associated with acute episodes of schizophrenia has not diminished in Germany over the last 30 years, but has become more severe. The stigma of depression has slightly improved, but not to the extent that could have been expected given the increasingly open discourse about mental health problems. Seemingly, there is no “trickle down” of de-stigmatization of common mental health problems to severe mental illness, particularly if psychotic symptoms are involved that seem incomprehensible and outside “normal” experiences. On the contrary, a growing familiarity with symptoms of depression, that enables taking the role of the affected person, might leave psychotic, incomprehensible symptoms look even more unrelatable. Antistigma efforts thus need to (re-)focus on severe, acute mental illness, including advocacy for a more responsible and comprehensive portrayal of schizophrenia in the media.

## Data Availability

The data that support these findings are available from the corresponding author upon reasonable request.

## Supplementary material

For supplementary material accompanying this paper visit https://doi.org/10.1192/j.eurpsy.2022.2337. click here to view supplementary material
