# Supplementary material for: Changes in mental illness stigma over 30 years – Improvement, persistence, or deterioration?
Source: Eur Psychiatry. 2022 Nov 4;65(1):e78. doi: 10.1192/j.eurpsy.2022.2337 (PMC9724218; doi:10.1192/j.eurpsy.2022.2337)
Supplement: Supplementary file 1 [file S0924933822023379sup001.docx]

**Changes in mental illness stigma over 30 years – improvement, persistence, or detorioration?**

Georg Schomerus, Stephanie Schindler, Christian Sander, Eva Baumann, Matthias C. Angermeyer

**Supplementary Material**

**Methods**

Sampling method

We used a three stage random sampling procedure with (a) sample points (electoral wards), (b) households, and (c) individuals within target households. Target households within sample points were determined according to the random route procedure, that is, a street was selected randomly as a starting point from which interviewers followed a set route through the area. Target individuals were selected using random digits. Informed consent was considered to have been given when individuals agreed to complete the interview. Fieldwork was carried out in 1990 by GETAS (Hamburg) and in 2001, 2011 and 2020 by USUMA (Berlin); both companies were specialized in market and social research.

Statistical analysis

All analyses were performed using Stata special edition 16.0. Given the study design of repeated cross-sections with random sampling and without clustering, we assumed the data to be independent observations without serial correlation. We therefore performed a multiple regression analysis for each individual 5-point item representing emotional reactions to, or the desire for social distance from people with schizophrenia and depression as criterion using an ordinary least squares estimator. Robust standard errors were computed to account for heteroscedasticity. Survey (1990, 2001, 201 and 2020), vignette (depression or schizophrenia), interaction of survey*vignette, age of respondents, and gender of the vignette or respondents, respectively, entered the analyses as regressors.

Hypothesis H1, predicting an improvement of attitudes over the past 30 years, was tested separately for both vignettes via contrasts comparing the first survey (1990) with the last (2020). All covariates were evaluated at the observed values in the sample.

We tested hypothesis H2, assuming an initial gap between both vignettes at the start of the observation period, by contrasting both vignettes at baseline (1990).

Hypothesis H3, predicting a divergence of both disorders from 1990 to 2020, was tested in terms of the interaction of vignette and survey 1990 versus 2020. The interaction term represents the effect superseding the additive effects of survey and vignette relative to 1990 (i.e., how much stronger or weaker was the time-related change for one vignette as compared to the change of the other vignette) and thus indicates a divergence instead of a parallel development of both vignettes.

To answer research question 3, that is, to identify critical periods, we used exploratory contrasts for each vignette that compared each follow-up survey (2001, 2011 and 2020) with the preceding one. Again, all covariates were evaluated at the observed values in the sample.

Respondents of the survey in 2020 who chose to answer the interview forms without interviewer were on average 3.9 years younger (t = -4.39, p < .001, two-sample t-test) and reported more years of education (χ² = 39.30, p < .001, Pearson Chi square test). We therefore validated all results against sensitivity analyses that excluded self-administered interviews.

All *p* values were corrected for separate testing of items, vignettes, and, where applicable, for the number of time periods using the Benjamini-Hochberg procedure to control the false discovery rate at *q* = 0.05.

**Case-vignettes (she)**

Schizophrenia

Please imagine that you find out the following about an acquaintance with whom you are occasionally doing something in your free time:

Within the past six months, your acquaintance appears to have changed. More and more, she retreated from her friends and colleagues, up to the point of avoiding them. If someone managed to involve her in a conversation, she would address only one single topic: the question as to whether some people had the natural gift of reading other people’s thoughts. This question became her sole concern. In contrast with her previous habits, she stopped taking care of her appearance and looked increasingly untidy. At work, she seemed absent-minded and frequently made mistakes. As a consequence, she has already been summoned to his boss.

Finally, your acquaintance stayed away from work for an entire week without an excuse. Upon her return, she seemed anxious and hounded. she reports that she is now absolutely certain, that people cannot only read other people’s thoughts, but that they also directly influence them. She was however unsure who would steer her thoughts. She also said that, when thinking, she was continually interrupted. Frequently, she would even hear those people talk to her, and they would give her instructions. Sometimes, they would also talk to each other and make fun of whatever she was doing at the time. The situation was particularly bad at her apartment, she claimed. At home, she would really feel threatened, and would be terribly scared. Hence she had not spent the night at her place for the past week, but rather she had hidden in hotel rooms and hardly dared to go out.

Major depressive disorder

Please imagine that you find out the following about an acquaintance with whom you are occasionally doing something in your free time:

Within the past two months, your acquaintance has changed in her nature. As opposed to previously, she is down and sad without being able to make out a tangible reason for her feeling low. She appears serious and worried. There is nothing anymore that will make her laugh. She hardly ever talks, and if she says something, she speaks in a low tone of voice about the worries she has with regard to her future. Your acquaintance feels useless and has the impression to do everything wrong. All attempts to cheer her up have failed. She lost all interest in things and is not motivated to do anything. She complains of often waking up in the middle of the night and not being able to get back to sleep. Already in the morning, she feels exhausted and without energy. She says that she encounters difficulty in concentrating on her job. In contrast with previous times, everything takes her very long. She hardly manages her workload. As a consequence, she has already been summoned to her boss.

**Supplementary Tables**

**Table S1: Socio-demographic characteristics of the population samples**

|  | 1990 | | 2001 | | 2011 | | 2020 | |
| --- | --- | --- | --- | --- | --- | --- | --- | --- |
|  | Survey | Total population | Survey | Total population | Survey | Total population | Survey | Total population |
| Gender  Male  Female  Divers | 45.5  54.5 | 48.5  51.5 | 43.9  56.1 | 48.3  51.7 | 45.4  54.6 | 48.6  51.4 | 45.3  54.2  0.5 | 49.3  50.7 |
| Age, years  18-25  26-45  46-60  61+ | 11.4  36.4  25.0  27.2 | 12.3  38.0  24.2  25.5 | 12.0  41.0  23.4  23.7 | 9.8  37.8  23.3  29.1 | 8.9  31.4  27.9  31.8 | 11.3  31.9  26.9  29.9 | 10.6  33.1  29.0  27.4 | 10.9  30.4  27.3  31.4 |
| Educational attainment  Still student  No schooling completed  8/9 years of schooling  10 years of schooling  12/13 years of schooling | 1.9  3.6  53.9  24.0  16.5 | 0.4  2.5  55.8  25.8  15.5 | 3.0  2.2  46.8  30.2  18.0 | 0.2  2.1  49.1  27.5  21.1 | 0.8  1.9  44.0  35.9  17.3 | 1.0  4.0  38.5  29.3  27.1 | 0.7  1.8  31.9  38.0  27.5 | 0.2  4.4  33.0  26.3  36.1 |

Percentages of sample/population. Population Data from the Federal Statistical Office of Germany

**Table S2: Predictors of the desire for social distancing from someone with schizophrenia or depression**

| Desire for social distance related to… (N) | Predictor | df | *F* | *p* |
| --- | --- | --- | --- | --- |
| Marriage into family (10377) | Period | 3 | 22.24 | <.001*** |
|  | Vignette (S; D) | 1 | 410.18 | <.001*** |
|  | Period*vignette | 3 | 20.36 | <.001*** |
|  | Gender of vignette (m; f) | 1 | 14.88 | <.001*** |
|  | Gender of respondent (m; f) | 1 | 19.49 | <.001*** |
|  | Age of respondent | 1 | 371.65 | <.001*** |
|  | Full model | 10 | 99.89 | <.001*** |
| Introduction to girlfriend (10379) | Period | 3 | 11.36 | <.001*** |
|  | Vignette (S; D) | 1 | 314.42 | <.001*** |
|  | Period*vignette | 3 | 14.20 | <.001*** |
|  | Gender of vignette (m; f) | 1 | 42.68 | <.001*** |
|  | Gender of respondent (m; f) | 1 | 32.09 | <.001*** |
|  | Age of respondent | 1 | 142.68 | <.001*** |
|  | Full model | 10 | 67.84 | <.001*** |
| Child care (10375) | Period | 3 | 31.07 | <.001*** |
|  | Vignette (S; D) | 1 | 522.14 | <.001*** |
|  | Period*vignette | 3 | 8.08 | <.001*** |
|  | Gender of vignette (m; f) | 1 | 9.00 | .003** |
|  | Gender of respondent (m; f) | 1 | 0.03 | .857 |
|  | Age of respondent | 1 | 33.95 | <.001*** |
|  | Full model | 10 | 76.44 | <.001*** |
| Subtenant (10377) | Period | 3 | 8.45 | <.001*** |
|  | Vignette (S; D) | 1 | 618.01 | <.001*** |
|  | Period*vignette | 3 | 20.06 | <.001*** |
|  | Gender of vignette (m; f) | 1 | 9.24 | .002** |
|  | Gender of respondent (m; f) | 1 | 1.45 | .228 |
|  | Age of respondent | 1 | 90.14 | <.001*** |
|  | Full model | 10 | 83.18 | <.001*** |
| Colleague (10373) | Period | 3 | 28.11 | <.001*** |
|  | Vignette (S; D) | 1 | 400.66 | <.001*** |
|  | Period*vignette | 3 | 18.03 | <.001*** |
|  | Gender of vignette (m; f) | 1 | 1.97 | .161 |
|  | Gender of respondent (m; f) | 1 | 22.38 | <.001*** |
|  | Age of respondent | 1 | 64.44 | <.001*** |
|  | Full model | 10 | 63.31 | <.001*** |
| Neighbor (10361) | Period | 3 | 16.73 | <.001*** |
|  | Vignette (S; D) | 1 | 483.36 | <.001*** |
|  | Period*vignette | 3 | 19.25 | <.001*** |
|  | Gender of vignette (m; f) | 1 | 2.04 | .154 |
|  | Gender of respondent (m; f) | 1 | 30.33 | <.001*** |
|  | Age of respondent | 1 | 89.94 | <.001*** |
|  | Full model | 10 | 73.53 | <.001*** |
| Work recommendation (10380) | Period | 3 | 21.81 | <.001*** |
|  | Vignette (S; D) | 1 | 341.08 | <.001*** |
|  | Period*vignette | 3 | 13.85 | <.001*** |
|  | Gender of vignette (m; f) | 1 | 4.88 | .027* |
|  | Gender of respondent (m; f) | 1 | 8.89 | .003** |
|  | Age of respondent | 1 | 85.82 | <.001*** |
|  | Full model | 10 | 57.15 | <.001*** |

Results of the multiple regression analyses of the desire for social distance from someone with schizophrenia or depression with period, vignette, interaction of period*vignette, age, and gender of vignette or respondents as predictors. The between-subjects effects are reported (*F* statistic) with uncorrected *p* values. *N* sample size. * *p*<.05, ** *p*<.01, *** *p*<.001.

**Table S3: Changes in the desire for social distance from someone with schizophrenia or depression, 1990-2020**

| Sample specifics | Desire for social distance related to… | Sample (*N*) | Vignette | Parameter estimates | | | Test statistics | |
| --- | --- | --- | --- | --- | --- | --- | --- | --- |
|  |  |  |  | *b* | SE | [95% CI] | *t* | *p* |
| Represen-tative West German population sample | Marriage into family | 10377 | Depression | -0.45 | 0.05 | -0.54, -0.35 | -9.16 | <.001*** |
|  |  |  | Schizophrenia | 0.00 | 0.05 | -0.10, 0.09 | -0.05 | .961 |
|  | Introduction to a friend | 10379 | Depression | -0.09 | 0.05 | -0.19, 0.01 | -1.71 | .122 |
|  |  |  | Schizophrenia | 0.34 | 0.05 | 0.24, 0.44 | 6.79 | <.001*** |
|  | Child care | 10375 | Depression | 0.11 | 0.05 | 0.01, 0.20 | 2.19 | .045* |
|  |  |  | Schizophrenia | 0.41 | 0.04 | 0.32, 0.49 | 9.40 | <.001*** |
|  | Subtenant | 10377 | Depression | -0.14 | 0.05 | -0.24, -0.05 | -2.92 | .006** |
|  |  |  | Schizophrenia | 0.33 | 0.05 | 0.23, 0.42 | 6.76 | <.001*** |
|  | Colleague | 10373 | Depression | 0.07 | 0.05 | -0.02, 0.16 | 1.46 | .177 |
|  |  |  | Schizophrenia | 0.50 | 0.05 | 0.41, 0.60 | 10.14 | <.001*** |
|  | Neighbor | 10361 | Depression | -0.06 | 0.05 | -0.15, 0.04 | -1.22 | .240 |
|  |  |  | Schizophrenia | 0.41 | 0.05 | 0.31, 0.51 | 8.35 | <.001*** |
|  | Work recommendation | 10380 | Depression | 0.07 | 0.05 | -0.03, 0.16 | 1.43 | .177 |
|  |  |  | Schizophrenia | 0.44 | 0.05 | 0.35, 0.53 | 9.24 | <.001*** |
| Sensitivity analysis | Marriage into family | 9926 | Depression | -0.39 | 0.05 | -0.49, -0.29 | -7.64 | <.001*** |
|  |  |  | Schizophrenia | 0.06 | 0.05 | -0.04, 0.16 | 1.22 | .239 |
|  | Introduction to a friend | 9930 | Depression | -0.08 | 0.05 | -0.19, 0.03 | -1.49 | .160 |
|  |  |  | Schizophrenia | 0.39 | 0.05 | 0.29, 0.50 | 7.40 | <.001*** |
|  | Child care | 9925 | Depression | 0.11 | 0.05 | 0.01, 0.21 | 2.18 | .046* |
|  |  |  | Schizophrenia | 0.40 | 0.05 | 0.31, 0.49 | 8.76 | <.001*** |
|  | Subtenant | 9926 | Depression | -0.13 | 0.05 | -0.23, -0.03 | -2.49 | .022* |
|  |  |  | Schizophrenia | 0.36 | 0.05 | 0.26, 0.46 | 7.02 | <.001*** |
|  | Colleague | 9922 | Depression | 0.10 | 0.05 | 0.00, 0.19 | 2.02 | .061 |
|  |  |  | Schizophrenia | 0.56 | 0.05 | 0.46, 0.66 | 10.62 | <.001*** |
|  | Neighbor | 9911 | Depression | -0.02 | 0.05 | -0.12, 0.08 | -0.44 | .656 |
|  |  |  | Schizophrenia | 0.47 | 0.05 | 0.37, 0.57 | 9.20 | <.001*** |
|  | Work recommendation | 9931 | Depression | 0.09 | 0.05 | 0.00, 0.19 | 1.88 | .077 |
|  |  |  | Schizophrenia | 0.46 | 0.05 | 0.37, 0.56 | 9.26 | <.001*** |

Selected contrasts of multiple regression analyses with the desire for social distance as criteria and period, vignette, interaction of period*vignette, age, and gender of vignette or respondents as predictors. The contrast estimates describe the change from 1990 to 2020 per vignette. Positive values indicate an increase in the desire for social distance. The sensitivity analyses exclude self-administered interviews, an option specific to 2020 due to the COVID-19 pandemic. The *p* values are corrected for separate testing of items and vignettes using the Benjamini-Hochberg procedure to control the false discovery rate at *q* = .05. * *p*<.05, ** *p*<.01, *** *p*<.001.

**Table S4: Predictors of emotional reactions to someone with schizophrenia or depression**

| Emotional reaction (*N*) | Predictor | df | *F* | *p* |
| --- | --- | --- | --- | --- |
| Fear (10383) | Period | 3 | 1.16 | .325 |
|  | Vignette (S; D) | 1 | 354.60 | <.001*** |
|  | Period*vignette | 3 | 8.37 | <.001*** |
|  | Gender of vignette (m; f) | 1 | 0.24 | .626 |
|  | Gender of respondent (m; f) | 1 | 79.24 | <.001*** |
|  | Age of respondent | 1 | 12.87 | <.001*** |
|  | Full model | 10 | 47.57 | <.001*** |
| Uneasiness (10379) | Period | 3 | 5.18 | .001** |
|  | Vignette (S; D) | 1 | 371.57 | <.001*** |
|  | Period*vignette | 3 | 16.55 | <.001*** |
|  | Gender of vignette (m; f) | 1 | 0.02 | .879 |
|  | Gender of respondent (m; f) | 1 | 12.61 | <.001*** |
|  | Age of respondent | 1 | 10.67 | .001** |
|  | Full model | 10 | 45.88 | <.001*** |
| Insecurity (10367) | Period | 3 | 4.36 | .005** |
|  | Vignette (S; D) | 1 | 205.93 | <.001*** |
|  | Period*vignette | 3 | 2.30 | .075 |
|  | Gender of vignette (m; f) | 1 | 3.73 | .054 |
|  | Gender of respondent (m; f) | 1 | 34.43 | <.001*** |
|  | Age of respondent | 1 | 5.20 | .023* |
|  | Full model | 10 | 28.16 | <.001*** |
| Anger (10369) | Period | 3 | 9.31 | <.001*** |
|  | Vignette (S; D) | 1 | 62.62 | <.001*** |
|  | Period*vignette | 3 | 0.48 | .698 |
|  | Gender of vignette (m; f) | 1 | 0.33 | .566 |
|  | Gender of respondent (m; f) | 1 | 43.36 | <.001*** |
|  | Age of respondent | 1 | 6.58 | .010* |
|  | Full model | 10 | 14.86 | <.001*** |
| Amusement (10373) | Period | 3 | 5.01 | .002** |
|  | Vignette (S; D) | 1 | 32.83 | <.001*** |
|  | Period*vignette | 3 | 0.96 | .411 |
|  | Gender of vignette (m; f) | 1 | 4.16 | .041* |
|  | Gender of respondent (m; f) | 1 | 45.89 | <.001*** |
|  | Age of respondent | 1 | 6.97 | .008** |
|  | Full model | 10 | 11.26 | <.001*** |
| Irritation (10367) | Period | 3 | 11.82 | <.001*** |
|  | Vignette (S; D) | 1 | 71.13 | <.001*** |
|  | Period*vignette | 3 | 5.35 | .001** |
|  | Gender of vignette (m; f) | 1 | 0.71 | .401 |
|  | Gender of respondent (m; f) | 1 | 51.43 | <.001*** |
|  | Age of respondent | 1 | 20.41 | <.001*** |
|  | Full model | 10 | 20.76 | <.001*** |
| Sympathy (10366) | Period | 3 | 7.21 | <.001*** |
|  | Vignette (S; D) | 1 | 139.71 | <.001*** |
|  | Period*vignette | 3 | 4.99 | .002** |
|  | Gender of vignette (m; f) | 1 | 12.34 | <.001*** |
|  | Gender of respondent (m; f) | 1 | 37.69 | <.001*** |
|  | Age of respondent | 1 | 0.87 | .352 |
|  | Full model | 10 | 21.76 | <.001*** |
| Compassion (10373) | Period | 3 | 14.88 | <.001*** |
|  | Vignette (S; D) | 1 | 3.17 | .075 |
|  | Period*vignette | 3 | 2.12 | .096 |
|  | Gender of vignette (m; f) | 1 | 9.02 | .003** |
|  | Gender of respondent (m; f) | 1 | 108.52 | <.001*** |
|  | Age of respondent | 1 | 2.23 | .135 |
|  | Full model | 10 | 17.43 | <.001*** |
| Desire to help (10372) | Period | 3 | 1.56 | .198 |
|  | Vignette (S; D) | 1 | 40.07 | <.001*** |
|  | Period*vignette | 3 | 11.25 | <.001*** |
|  | Gender of vignette (m; f) | 1 | 5.42 | .020* |
|  | Gender of respondent (m; f) | 1 | 192.45 | <.001*** |
|  | Age of respondent | 1 | 10.86 | .001** |
|  | Full model | 10 | 29.14 | <.001*** |
| Lack of understanding (10372) | Period | 3 | 10.20 | <.001*** |
|  | Vignette (S; D) | 1 | 139.65 | <.001*** |
|  | Period*vignette | 3 | 1.98 | .115 |
|  | Gender of vignette (m; f) | 1 | 0.15 | .702 |
|  | Gender of respondent (m; f) | 1 | 52.42 | <.001*** |
|  | Age of respondent | 1 | 12.73 | <.001*** |
|  | Full model | 10 | 26.39 | <.001*** |

Results of the multiple regression analyses of emotional reactions towards someone with schizophrenia or depression with period, vignette, interaction of period*vignette, age, and gender of vignette or respondents as predictors. The between-subjects effects are reported (*F* statistic) with uncorrected *p* values. *N* sample size. * *p*<.05, ** *p*<.01, *** *p*<.001.

**Table S5: Changes in emotional reactions toward someone with schizophrenia or depression from 1990 to 2020**

| Sample specifics | Emotional reaction | Sample (*N*) | Vignette | Parameter estimates | | | Test statistics | |
| --- | --- | --- | --- | --- | --- | --- | --- | --- |
|  |  |  |  | *b* | SE | [95% CI] | *t* | *p* |
| Represen-tative West German population sample | Fear | 10383 | Depression | -0.07 | 0.05 | -0.18, 0.04 | -1.27 | .254 |
|  |  |  | Schizophrenia | 0.20 | 0.06 | 0.09, 0.31 | 3.55 | .001** |
|  | Uneasiness | 10379 | Depression | -0.29 | 0.05 | -0.40, -0.19 | -5.33 | <.001*** |
|  |  |  | Schizophrenia | 0.20 | 0.05 | 0.09, 0.30 | 3.67 | <.001*** |
|  | Insecurity | 10367 | Depression | -0.08 | 0.05 | -0.18, 0.02 | -1.57 | .163 |
|  |  |  | Schizophrenia | 0.11 | 0.05 | 0.01, 0.22 | 2.08 | .063 |
|  | Anger | 10369 | Depression | -0.07 | 0.04 | -0.15, 0.01 | -1.69 | .140 |
|  |  |  | Schizophrenia | -0.04 | 0.04 | -0.13, 0.04 | -0.99 | .360 |
|  | Amusement | 10373 | Depression | -0.09 | 0.03 | -0.16, -0.03 | -2.81 | .011* |
|  |  |  | Schizophrenia | -0.03 | 0.04 | -0.10, 0.04 | -0.73 | .487 |
|  | Irritation | 10367 | Depression | -0.13 | 0.04 | -0.22, -0.04 | -2.86 | .011* |
|  |  |  | Schizophrenia | 0.03 | 0.05 | -0.06, 0.12 | 0.67 | .502 |
|  | Sympathy | 10366 | Depression | 0.18 | 0.05 | 0.09, 0.28 | 3.76 | .001** |
|  |  |  | Schizophrenia | -0.07 | 0.05 | -0.16, 0.02 | -1.55 | .163 |
|  | Compassion | 10373 | Depression | 0.12 | 0.05 | 0.03, 0.21 | 2.55 | .022* |
|  |  |  | Schizophrenia | 0.17 | 0.05 | 0.08, 0.27 | 3.78 | <.001*** |
|  | Desire to help | 10372 | Depression | 0.11 | 0.05 | 0.02, 0.20 | 2.33 | .036* |
|  |  |  | Schizophrenia | -0.20 | 0.05 | -0.29, -0.11 | -4.33 | <.001*** |
|  | Lack of understanding | 10372 | Depression | -0.18 | 0.05 | -0.28, -0.08 | -3.68 | <.001*** |
|  |  |  | Schizophrenia | -0.06 | 0.05 | -0.16, 0.05 | -1.09 | .325 |
| Sensitivity analysis | Fear | 9932 | Depression | -0.07 | 0.06 | -0.18, 0.05 | -1.18 | .318 |
|  |  |  | Schizophrenia | 0.27 | 0.06 | 0.15, 0.38 | 4.52 | .001** |
|  | Uneasiness | 9929 | Depression | -0.27 | 0.06 | -0.38, -0.15 | -4.62 | <.001*** |
|  |  |  | Schizophrenia | 0.28 | 0.06 | 0.17, 0.39 | 4.95 | <.001*** |
|  | Insecurity | 9917 | Depression | -0.03 | 0.06 | -0.14, 0.08 | -0.59 | .614 |
|  |  |  | Schizophrenia | 0.22 | 0.06 | 0.11, 0.34 | 3.87 | <.001*** |
|  | Anger | 9920 | Depression | -0.06 | 0.04 | -0.15, 0.02 | -1.52 | .197 |
|  |  |  | Schizophrenia | -0.03 | 0.05 | -0.12, 0.06 | -0.65 | .605 |
|  | Amusement | 9922 | Depression | -0.07 | 0.03 | -0.14, -0.01 | -2.17 | .055 |
|  |  |  | Schizophrenia | -0.02 | 0.04 | -0.10, 0.05 | -0.55 | .614 |
|  | Irritation | 9917 | Depression | -0.09 | 0.05 | -0.18, 0.00 | -1.87 | .102 |
|  |  |  | Schizophrenia | 0.07 | 0.05 | -0.03, 0.17 | 1.36 | .250 |
|  | Sympathy | 9917 | Depression | 0.14 | 0.05 | 0.04, 0.24 | 2.72 | .017* |
|  |  |  | Schizophrenia | -0.13 | 0.05 | -0.23, -0.04 | -2.70 | .017* |
|  | Compassion | 9924 | Depression | 0.12 | 0.05 | 0.02, 0.21 | 2.38 | .037* |
|  |  |  | Schizophrenia | 0.16 | 0.05 | 0.06, 0.25 | 3.24 | .004** |
|  | Desire to help | 9920 | Depression | 0.06 | 0.05 | -0.04, 0.15 | 1.13 | .321 |
|  |  |  | Schizophrenia | -0.29 | 0.05 | -0.38, -0.19 | -5.97 | <.001*** |
|  | Lack of understanding | 9921 | Depression | -0.12 | 0.05 | -0.22, -0.02 | -2.35 | .037* |
|  |  |  | Schizophrenia | 0.01 | 0.06 | -0.10, 0.12 | 0.20 | .844 |

Selected contrasts of multiple regression analyses with emotional reactions as criteria and period, vignette, interaction of period*vignette, age, and gender of vignette or respondents as predictors. The contrast estimates describe the change from1990 to 2020 per vignette. Positive values indicate stronger emotional reactions. The sensitivity analyses exclude self-administered interviews, an option specific to 2020 due to the COVID-19 pandemic. The *p* values are corrected for separate testing of items and vignettes using the Benjamini-Hochberg procedure to control the false discovery rate at *q* = .05. * *p*<.05, ** *p*<.01, *** *p*<.001.

**Table S6: Sensitivity analyses for the differences in the desire for social distance from someone with schizophrenia or depression, 1990-2020**

| Desire for social distance related to… | Sample (*N*) | Parameter | Estimates | | | Test statistics | |
| --- | --- | --- | --- | --- | --- | --- | --- |
|  |  |  | *b* | SE (robust) | [95% CI] | *t* | *p* |
| Marriage into family | 9926 | Vignette (1990) | 0.11 | 0.05 | 0.01, 0.20 | 2.12 | .034* |
|  |  | 2020*vignette | 0.45 | 0.07 | 0.31, 0.59 | 6.28 | <.001*** |
| Introduction to friend | 9930 | Vignette (1990) | 0.13 | 0.05 | 0.02, 0.23 | 2.39 | .020* |
|  |  | 2020*vignette | 0.47 | 0.08 | 0.33, 0.62 | 6.27 | <.001*** |
| Child care | 9925 | Vignette (1990) | 0.27 | 0.05 | 0.17, 0.37 | 5.31 | <.001*** |
|  |  | 2020*vignette | 0.28 | 0.07 | 0.15, 0.42 | 4.15 | <.001*** |
| Subtenant | 9926 | Vignette (1990) | 0.22 | 0.05 | 0.12, 0.32 | 4.39 | <.001*** |
|  |  | 2020*vignette | 0.49 | 0.07 | 0.35, 0.63 | 6.68 | <.001*** |
| Colleague | 9922 | Vignette (1990) | 0.13 | 0.05 | 0.03, 0.22 | 2.49 | .020* |
|  |  | 2020*vignette | 0.46 | 0.07 | 0.32, 0.60 | 6.40 | <.001*** |
| Neighbor | 9911 | Vignette (1990) | 0.16 | 0.05 | 0.06, 0.26 | 3.21 | .003** |
|  |  | 2020*vignette | 0.50 | 0.07 | 0.36, 0.64 | 6.92 | <.001*** |
| Work recom-mendation | 9931 | Vignette (1990) | 0.12 | 0.05 | 0.02, 0.22 | 2.41 | .020* |
|  |  | 2020*vignette | 0.37 | 0.07 | 0.23, 0.51 | 5.19 | <.001*** |

Results of multiple regression analyses of the items of the Desire for Social Distance Scale with period, vignette, interaction of period*vignette, age, and gender of vignette or respondents as predictors, excluding respondents with self-administered interviews in 2020 from the analyses. We show selected estimates (the initial gap between schizophrenia and depression in 1990, and the increase of this gap until 2020) with *p* values corrected for separate testing of items using the Benjamini-Hochberg procedure to control the false discovery rate at *q* = .05. * *p*<.05, ** *p*<.01, *** *p*<.001.

**Table S7: Sensitivity analyses for differences in emotional reactions to someone with schizophrenia or depression, 1990-2020**

| Emotional reaction | Sample (*N*) | Parameter | Estimates | | | Test statistics | |
| --- | --- | --- | --- | --- | --- | --- | --- |
|  |  |  | *b* | SE (robust) | [95% CI] | *t* | *p* |
| Fear | 9932 | Vignette (1990) | 0.25 | 0.06 | 0.14, 0.36 | 4.40 | <.001*** |
|  |  | 2020*vignette | 0.33 | 0.08 | 0.17, 0.50 | 4.06 | <.001*** |
| Uneasiness | 9929 | Vignette (1990) | 0.15 | 0.06 | 0.04, 0.27 | 2.73 | .010* |
|  |  | 2020*vignette | 0.55 | 0.08 | 0.39, 0.70 | 6.77 | <.001*** |
| Insecurity | 9917 | Vignette (1990) | 0.23 | 0.06 | 0.12, 0.34 | 4.15 | <.001*** |
|  |  | 2020*vignette | 0.26 | 0.08 | 0.10, 0.41 | 3.21 | .003** |
| Anger | 9920 | Vignette (1990) | 0.16 | 0.04 | 0.07, 0.24 | 3.55 | .001** |
|  |  | 2020*vignette | 0.03 | 0.06 | -0.09, 0.16 | 0.55 | .584 |
| Amusement | 9922 | Vignette (1990) | 0.06 | 0.04 | -0.01, 0.14 | 1.75 | .090 |
|  |  | 2020*vignette | 0.05 | 0.05 | -0.05, 0.15 | 1.05 | .367 |
| Irritation | 9917 | Vignette (1990) | 0.16 | 0.05 | 0.07, 0.25 | 3.35 | .002** |
|  |  | 2020*vignette | 0.16 | 0.07 | 0.02, 0.29 | 2.27 | .039* |
| Sympathy | 9917 | Vignette (1990) | -0.11 | 0.05 | -0.21, -0.01 | -2.27 | .029* |
|  |  | 2020*vignette | -0.27 | 0.07 | -0.41, -0.13 | -3.84 | <.001*** |
| Compassion | 9924 | Vignette (1990) | -0.06 | 0.05 | -0.15, 0.04 | -1.17 | .242 |
|  |  | 2020*vignette | 0.04 | 0.07 | -0.09, 0.18 | 0.60 | .584 |
| Desire to help | 9920 | Vignette (1990) | 0.11 | 0.05 | 0.02, 0.21 | 2.35 | .027* |
|  |  | 2020*vignette | -0.35 | 0.07 | -0.48, -0.21 | -4.98 | <.001*** |
| Lack of understanding | 9921 | Vignette (1990) | 0.25 | 0.05 | 0.14, 0.36 | 4.64 | <.001*** |
|  |  | 2020*vignette | 0.13 | 0.08 | -0.02, 0.28 | 1.74 | .118 |

Results of multiple regression analyses of the items of the Emotional Reactions towards Mental Illness Scale, with period, vignette, interaction of period*vignette, age, and gender of vignette or respondents as predictors, and excluding respondents with self-administered interviews in 2020 from the analyses. We show selected estimates (the initial gap between schizophrenia and depression in 1990, and the increase of this gap between until 2020) with *p* values corrected for separate testing of items using the Benjamini-Hochberg procedure to control the false discovery rate at *q* = .05. * *p*<.05, ** *p*<.01, *** *p*<.001.

**Table S8: Changes in the desire for social distance from someone with schizophrenia or depression by decade, 1990-2001, 2001-2011, 2011-2020.**

| Desire for social distance related to… | Sample specifics and size (*N*) | Parameter | Estimates | | | Test statistics | | |
| --- | --- | --- | --- | --- | --- | --- | --- | --- |
|  |  |  | *b* | SE | [95% CI] | *t* | *q* |  |
| Marriage into family | Represen-tative West German population sample (10377) | Depression 2001-1990 | -0.19 | 0.05 | -0.28, -0.10 | -4.03 | <.001*** |  |
|  |  | Depression 2011-2001 | -0.13 | 0.05 | -0.23, -0.04 | -2.79 | .017* |  |
|  |  | Depression 2020-2011 | -0.12 | 0.05 | -0.22, -0.03 | -2.52 | .033* |  |
|  |  | Schizophrenia 2001-1990 | 0.23 | 0.04 | 0.14, 0.31 | 5.03 | <.001*** |  |
|  |  | Schizophrenia 2011-2001 | -0.09 | 0.05 | -0.18, 0.00 | -1.91 | .132 |  |
|  |  | Schizophrenia 2020-2011 | -0.14 | 0.05 | -0.24,-0.04 | -2.87 | .014* |  |
|  | Sensitivity analysis (9926) | Depression 2001-1990 | -0.19 | 0.05 | -0.28, -0.10 | -4.05 | <.001*** |  |
|  |  | Depression 2011-2001 | -0.13 | 0.05 | -0.22, -0.04 | -2.73 | .019* |  |
|  |  | Depression 2020-2011 | -0.07 | 0.05 | -0.17, 0.03 | -1.37 | .277 |  |
|  |  | Schizophrenia 2001-1990 | 0.22 | 0.05 | 0.14, 0.31 | 4.97 | <.001*** |  |
|  |  | Schizophrenia 2011-2001 | -0.08 | 0.05 | -0.17, 0.01 | -1.84 | .144 |  |
|  |  | Schizophrenia 2020-2011 | -0.08 | 0.05 | -0.18,0.02 | -1.50 | .227 |  |
| Introduction to girlfriend | Represen-tative West German population sample (10379) | Depression 2001-1990 | 0.04 | 0.05 | -0.06, 0.13 | 0.73 | .543 |  |
|  |  | Depression 2011-2001 | -0.05 | 0.05 | -0.15, 0.05 | -1.06 | .390 |  |
|  |  | Depression 2020-2011 | -0.07 | 0.05 | -0.17, 0.03 | -1.33 | .296 |  |
|  |  | Schizophrenia 2001-1990 | 0.36 | 0.05 | 0.26, 0.45 | 7.31 | <.001*** |  |
|  |  | Schizophrenia 2011-2001 | 0.03 | 0.05 | -0.07. 0.13 | 0.53 | .639 |  |
|  |  | Schizophrenia 2020-2011 | -0.04 | 0.05 | -0.14, 0.06 | -0.78 | .524 |  |
|  | Sensitivity analysis (9930) | Depression 2001-1990 | 0.04 | 0.05 | -0.06, 0.14 | 0.75 | .530 |  |
|  |  | Depression 2011-2001 | -0.06 | 0.05 | -0.16, 0.05 | -1.08 | .378 |  |
|  |  | Depression 2020-2011 | -0.06 | 0.05 | -0.17, 0.05 | -1.14 | .357 |  |
|  |  | Schizophrenia 2001-1990 | 0.36 | 0.05 | 0.26, 0.45 | 7.30 | <.001*** |  |
|  |  | Schizophrenia 2011-2001 | 0.03 | 0.05 | -0.07. 0.13 | 0.51 | .657 |  |
|  |  | Schizophrenia 2020-2011 | 0.01 | 0.06 | -0.10, 0.12 | 0.19 | .850 |  |
| Child care | Represen-tative West German population sample (10375) | Depression 2001-1990 | 0.18 | 0.05 | 0.09. 0.27 | 3.74 | <.001*** |  |
|  |  | Depression 2011-2001 | -0.09 | 0.05 | -0.18, 0.01 | -1.80 | .151 |  |
|  |  | Depression 2020-2011 | 0.01 | 0.05 | -0.08, 0.11 | 0.28 | .816 |  |
|  |  | Schizophrenia 2001-1990 | 0.43 | 0.04 | 0.35, 0.51 | 10.15 | <.001*** |  |
|  |  | Schizophrenia 2011-2001 | -0.07 | 0.04 | -0.15, 0.02 | -1.57 | .212 |  |
|  |  | Schizophrenia 2020-2011 | 0.04 | 0.04 | -0.04, 0.13 | 0.99 | .410 |  |
|  | Sensitivity analysis (9925) | Depression 2001-1990 | 0.17 | 0.05 | 0.08. 0.27 | 3.60 | .001** |  |
|  |  | Depression 2011-2001 | -0.08 | 0.05 | -0.17, 0.01 | -1.65 | .197 |  |
|  |  | Depression 2020-2011 | 0.02 | 0.05 | -0.08, 0.12 | 0.35 | .746 |  |
|  |  | Schizophrenia 2001-1990 | 0.42 | 0.04 | 0.34, 0.51 | 9.96 | <.001*** |  |
|  |  | Schizophrenia 2011-2001 | -0.06 | 0.04 | -0.14, 0.02 | -1.40 | .259 |  |
|  |  | Schizophrenia 2020-2011 | 0.03 | 0.04 | -0.05, 0.12 | 0.75 | .530 |  |
| Subtenant | Represen-tative West German population sample (10377) | Depression 2001-1990 | -0.05 | 0.05 | -0.14, 0.04 | -1.03 | .398 |  |
|  |  | Depression 2011-2001 | -0.17 | 0.05 | -0.26, -0.07 | -3.32 | .003** |  |
|  |  | Depression 2020-2011 | 0.07 | 0.05 | -0.03, 0.17 | 1.39 | .279 |  |
|  |  | Schizophrenia 2001-1990 | 0.36 | 0.05 | 0.27, 0.45 | 7.75 | <.001*** |  |
|  |  | Schizophrenia 2011-2001 | -0.12 | 0.05 | -0.22, -0.02 | -2.46 | .037* |  |
|  |  | Schizophrenia 2020-2011 | 0.09 | 0.05 | -0.01, 0.19 | 1.71 | .175 |  |
|  | Sensitivity analysis (9926) | Depression 2001-1990 | -0.05 | 0.05 | -0.14, 0.05 | -0.96 | .431 |  |
|  |  | Depression 2011-2001 | -0.17 | 0.05 | -0.27, -0.07 | -3.38 | .003** |  |
|  |  | Depression 2020-2011 | 0.09 | 0.05 | -0.02, 0.19 | 1.57 | .213 |  |
|  |  | Schizophrenia 2001-1990 | 0.37 | 0.05 | 0.27, 0.46 | 7.79 | <.001*** |  |
|  |  | Schizophrenia 2011-2001 | -0.13 | 0.05 | -0.22, -0.03 | -2.51 | .031* |  |
|  |  | Schizophrenia 2020-2011 | 0.12 | 0.05 | 0.01, 0.22 | 2.21 | .063 |  |
| Colleague | Represen-tative West German population sample (10373) | Depression 2001-1990 | 0.06 | 0.05 | -0.03, 0.15 | 1.29 | .299 |  |
|  |  | Depression 2011-2001 | -0.04 | 0.05 | -0.13, 0.05 | -0.93 | .438 |  |
|  |  | Depression 2020-2011 | 0.05 | 0.05 | -0.04, 0.15 | 1.10 | .379 |  |
|  |  | Schizophrenia 2001-1990 | 0.47 | 0.05 | 0.37, 0.56 | 9.69 | <.001*** |  |
|  |  | Schizophrenia 2011-2001 | -0.06 | 0.05 | -0.16, 0.04 | -1.14 | .370 |  |
|  |  | Schizophrenia 2020-2011 | 0.09 | 0.05 | -0.01, 0.20 | 1.80 | .151 |  |
|  | Sensitivity analysis (9922) | Depression 2001-1990 | 0.06 | 0.05 | -0.03, 0.15 | 1.30 | .292 |  |
|  |  | Depression 2011-2001 | -0.04 | 0.05 | -0.14, 0.05 | -0.93 | .434 |  |
|  |  | Depression 2020-2011 | 0.08 | 0.05 | -0.02, 0.18 | 1.66 | .197 |  |
|  |  | Schizophrenia 2001-1990 | 0.47 | 0.05 | 0.37, 0.56 | 9.66 | <.001*** |  |
|  |  | Schizophrenia 2011-2001 | -0.06 | 0.05 | -0.16, 0.04 | -1.15 | .357 |  |
|  |  | Schizophrenia 2020-2011 | 0.15 | 0.06 | 0.04, 0.26 | 2.71 | .019* |  |
| Neighbor | Represen-tative West German population sample (10361) | Depression 2001-1990 | 0.03 | 0.05 | -0.06, 0.12 | 0.61 | .612 |  |
|  |  | Depression 2011-2001 | -0.16 | 0.05 | -0.25, -0.07 | -3.56 | .002** |  |
|  |  | Depression 2020-2011 | 0.08 | 0.05 | -0.02, 0.17 | 1.62 | .202 |  |
|  |  | Schizophrenia 2001-1990 | 0.42 | 0.05 | 0.33, 0.52 | 8.80 | <.001*** |  |
|  |  | Schizophrenia 2011-2001 | -0.14 | 0.05 | -0.23, -0.04 | -2.73 | .019* |  |
|  |  | Schizophrenia 2020-2011 | 0.12 | 0.05 | 0.02, 0.22 | 2.42 | .039* |  |
|  | Sensitivity analysis (9911) | Depression 2001-1990 | 0.03 | 0.05 | -0.06, 0.12 | 0.71 | .530 |  |
|  |  | Depression 2011-2001 | -0.17 | 0.05 | -0.25, -0.08 | -3.64 | .001** |  |
|  |  | Depression 2020-2011 | 0.11 | 0.05 | 0.01, 0.21 | 2.25 | .061 |  |
|  |  | Schizophrenia 2001-1990 | 0.43 | 0.05 | 0.33, 0.52 | 8.87 | <.001*** |  |
|  |  | Schizophrenia 2011-2001 | -0.14 | 0.05 | -0.24, -0.04 | -2.81 | .016* |  |
|  |  | Schizophrenia 2020-2011 | 0.19 | 0.05 | 0.08, 0.29 | 3.49 | .002** |  |
| Work recommend-dation | Represen-tative West German population sample (10380) | Depression 2001-1990 | -0.01 | 0.05 | -0.10, 0.08 | -0.21 | .834 |  |
|  |  | Depression 2011-2001 | 0.07 | 0.05 | -0.03, 0.16 | 1.41 | .278 |  |
|  |  | Depression 2020-2011 | 0.01 | 0.05 | -0.08, 0.11 | 0.25 | .824 |  |
|  |  | Schizophrenia 2001-1990 | 0.35 | 0.05 | 0.26, 0.44 | 7.63 | <.001*** |  |
|  |  | Schizophrenia 2011-2001 | 0.06 | 0.05 | -0.03, 0.15 | 1.28 | .299 |  |
|  |  | Schizophrenia 2020-2011 | 0.03 | 0.05 | -0.06, 0.12 | 0.59 | .612 |  |
|  | Sensitivity analysis (9931) | Depression 2001-1990 | -0.02 | 0.05 | -0.11, 0.07 | -0.40 | .722 |  |
|  |  | Depression 2011-2001 | 0.08 | 0.05 | -0.02, 0.17 | 1.61 | .204 |  |
|  |  | Depression 2020-2011 | 0.04 | 0.05 | -0.06, 0.14 | 0.73 | .530 |  |
|  |  | Schizophrenia 2001-1990 | 0.34 | 0.05 | 0.25, 0.43 | 7.38 | <.001*** |  |
|  |  | Schizophrenia 2011-2001 | 0.07 | 0.05 | -0.02, 0.16 | 1.50 | .227 |  |
|  |  | Schizophrenia 2020-2011 | 0.05 | 0.05 | -0.04, 0.15 | 1.06 | .378 |  |

Contrasts of multiple regression analyses with the items of the Desire for Social Distance Scale as criterion and period, vignette, interaction of period*vignette, age, and gender of vignette or respondents as predictors. The contrast estimates describe the change per period and vignette. Positive values indicate an increase in the desire for social distance. The sensitivity analyses exclude self-administered interviews, an option specific to 2020 due to the COVID-19 pandemic. *p* values are corrected for separate testing of items, periods, and vignettes using the Benjamini-Hochberg procedure to control the false discovery rate at *q* = .05. * *p*<.05, ** *p*<.01, *** *p*<.001.

**Table S9: Changes in emotional reactions towards someone with schizophrenia or depression by decade, 1990-2001, 2001-2011, 2011-2020.**

| Emotional reaction | Sample specifics and size (*N*) | Parameter | Estimates | | | Test statistics | |
| --- | --- | --- | --- | --- | --- | --- | --- |
|  |  |  | *b* | SE | [95% CI] | *t* | *p* |
| Fear | Represen-tative West German population sample (10383) | Depression 2001-1990 | -0.08 | 0.05 | -0.18, 0.01 | -1.67 | .171 |
|  |  | Depression 2011-2001 | -0.10 | 0.05 | -0.20, -0.01 | -2.09 | .095 |
|  |  | Depression 2020-2011 | 0.12 | 0.05 | 0.01, 0.23 | 2.24 | .076 |
|  |  | Schizophrenia 2001-1990 | 0.15 | 0.05 | 0.04, 0.25 | 2.79 | .025* |
|  |  | Schizophrenia 2011-2001 | 0.05 | 0.05 | -0.05, 0.16 | 1.01 | .466 |
|  |  | Schizophrenia 2020-2011 | -0.00 | 0.06 | -0.11, 0.11 | -0.03 | .980 |
|  | Sensitivity analysis (9932) | Depression 2001-1990 | -0.08 | 0.05 | -0.18, 0.02 | -1.59 | .208 |
|  |  | Depression 2011-2001 | -0.11 | 0.05 | -0.21, -0.01 | -2.16 | .079 |
|  |  | Depression 2020-2011 | 0.12 | 0.06 | 0.01, 0.23 | 2.15 | .079 |
|  |  | Schizophrenia 2001-1990 | 0.15 | 0.05 | 0.05, 0.25 | 2.86 | .020* |
|  |  | Schizophrenia 2011-2001 | 0.05 | 0.05 | -0.05, 0.16 | 0.94 | .456 |
|  |  | Schizophrenia 2020-2011 | 0.07 | 0.06 | -0.05, 0.18 | 1.10 | .397 |
| Uneasiness | Represen-tative West German population sample (10379) | Depression 2001-1990 | -0.07 | 0.05 | -0.17, 0.03 | -1.33 | .290 |
|  |  | Depression 2011-2001 | -0.17 | 0.05 | -0.28, -0.07 | -3.24 | .007** |
|  |  | Depression 2020-2011 | -0.05 | 0.06 | -0.16, 0.06 | -0.95 | .488 |
|  |  | Schizophrenia 2001-1990 | 0.26 | 0.05 | 0.15, 0.36 | 4.99 | <.001*** |
|  |  | Schizophrenia 2011-2001 | -0.02 | 0.05 | -0.13, 0.08 | -0.45 | .735 |
|  |  | Schizophrenia 2020-2011 | -0.03 | 0.05 | -0.14, 0.07 | -0.61 | .650 |
|  | Sensitivity analysis (9929) | Depression 2001-1990 | -0.06 | 0.05 | -0.16, 0.04 | -1.21 | .357 |
|  |  | Depression 2011-2001 | -0.18 | 0.05 | -0.28, -0.07 | -3.32 | .006** |
|  |  | Depression 2020-2011 | -0.03 | 0.06 | -0.14, 0.09 | -0.46 | .709 |
|  |  | Schizophrenia 2001-1990 | 0.26 | 0.05 | 0.16, 0.36 | 5.10 | <.001*** |
|  |  | Schizophrenia 2011-2001 | -0.03 | 0.05 | -0.13, 0.07 | -0.55 | .657 |
|  |  | Schizophrenia 2020-2011 | 0.05 | 0.06 | -0.06, 0.16 | 0.84 | .493 |
| Insecurity | Represen-tative West German population sample (10367) | Depression 2001-1990 | 0.02 | 0.05 | -0.07, 0.12 | 0.49 | .721 |
|  |  | Depression 2011-2001 | -0.14 | 0.05 | -0.24, -0.05 | -2.91 | .018* |
|  |  | Depression 2020-2011 | 0.04 | 0.05 | -0.06, 0.14 | 0.70 | .607 |
|  |  | Schizophrenia 2001-1990 | 0.14 | 0.05 | 0.04, 0.24 | 2.70 | .030* |
|  |  | Schizophrenia 2011-2001 | -0.12 | 0.05 | -0.22, -0.02 | -2.40 | .055 |
|  |  | Schizophrenia 2020-2011 | 0.10 | 0.05 | -0.01, 0.20 | 1.81 | .152 |
|  | Sensitivity analysis (9917) | Depression 2001-1990 | 0.03 | 0.05 | -0.07, 0.13 | 0.60 | .634 |
|  |  | Depression 2011-2001 | -0.15 | 0.05 | -0.24, -0.05 | -3.00 | .014* |
|  |  | Depression 2020-2011 | 0.08 | 0.05 | -0.02, 0.19 | 1.56 | .209 |
|  |  | Schizophrenia 2001-1990 | 0.14 | 0.05 | 0.04, 0.24 | 2.81 | .021* |
|  |  | Schizophrenia 2011-2001 | -0.13 | 0.05 | -0.22, -0.03 | -2.50 | .042* |
|  |  | Schizophrenia 2020-2011 | 0.21 | 0.06 | 0.09, 0.32 | 3.63 | .003** |
| Anger | Represen-tative West German population sample (10369) | Depression 2001-1990 | 0.08 | 0.04 | 0.01, 0.16 | 2.11 | .095 |
|  |  | Depression 2011-2001 | -0.01 | 0.04 | -0.09, 0.07 | -0.26 | .839 |
|  |  | Depression 2020-2011 | -0.14 | 0.04 | -0.23, -0.06 | -3.34 | .006** |
|  |  | Schizophrenia 2001-1990 | 0.10 | 0.04 | 0.02, 0.18 | 2.34 | .060 |
|  |  | Schizophrenia 2011-2001 | -0.07 | 0.04 | -0.15, 0.02 | -1.59 | .191 |
|  |  | Schizophrenia 2020-2011 | -0.07 | 0.05 | -0.16, 0.02 | -1.62 | .188 |
|  | Sensitivity analysis (9920) | Depression 2001-1990 | 0.09 | 0.04 | 0.01, 0.17 | 2.23 | .070 |
|  |  | Depression 2011-2001 | -0.02 | 0.04 | -0.10, 0.07 | -0.39 | .746 |
|  |  | Depression 2020-2011 | -0.14 | 0.04 | -0.22, -0.05 | -3.11 | .010* |
|  |  | Schizophrenia 2001-1990 | 0.10 | 0.04 | 0.02, 0.19 | 2.47 | .042* |
|  |  | Schizophrenia 2011-2001 | -0.07 | 0.04 | -0.16, 0.01 | -1.72 | .166 |
|  |  | Schizophrenia 2020-2011 | -0.06 | 0.05 | -0.15, 0.03 | -1.26 | .338 |
| Amusement | Represen-tative West German population sample (10373) | Depression 2001-1990 | 0.02 | 0.03 | -0.05, 0.08 | 0.50 | .721 |
|  |  | Depression 2011-2001 | -0.03 | 0.03 | -0.09, 0.04 | -0.80 | .580 |
|  |  | Depression 2020-2011 | -0.08 | 0.03 | -0.14, -0.02 | -2.56 | .039* |
|  |  | Schizophrenia 2001-1990 | 0.02 | 0.03 | -0.04, 0.09 | 0.72 | .599 |
|  |  | Schizophrenia 2011-2001 | 0.02 | 0.04 | -0.05, 0.09 | 0.44 | .736 |
|  |  | Schizophrenia 2020-2011 | -0.07 | 0.04 | -0.14, 0.01 | -1.76 | .155 |
|  | Sensitivity analysis (9922) | Depression 2001-1990 | 0.02 | 0.03 | -0.05, 0.09 | 0.60 | .634 |
|  |  | Depression 2011-2001 | -0.03 | 0.03 | -0.09, 0.03 | -0.91 | .461 |
|  |  | Depression 2020-2011 | -0.06 | 0.03 | -0.13, 0.00 | -1.92 | .121 |
|  |  | Schizophrenia 2001-1990 | 0.03 | 0.03 | -0.04, 0.09 | 0.84 | .493 |
|  |  | Schizophrenia 2011-2001 | 0.01 | 0.04 | -0.06, 0.08 | 0.32 | .758 |
|  |  | Schizophrenia 2020-2011 | -0.06 | 0.04 | -0.14, 0.02 | -1.53 | .215 |
| Irritation | Represen-tative West German population sample (10367) | Depression 2001-1990 | 0.16 | 0.04 | 0.07, 0.24 | 3.68 | .003** |
|  |  | Depression 2011-2001 | -0.19 | 0.04 | -0.28, -0.11 | -4.50 | <.001*** |
|  |  | Depression 2020-2011 | -0.09 | 0.04 | -0.18, -0.01 | -2.08 | .095 |
|  |  | Schizophrenia 2001-1990 | 0.10 | 0.04 | 0.01, 0.18 | 2.14 | .093 |
|  |  | Schizophrenia 2011-2001 | -0.08 | 0.04 | -0.17, 0.01 | -1.83 | .152 |
|  |  | Schizophrenia 2020-2011 | 0.02 | 0.05 | -0.07, 0.11 | 0.40 | .738 |
|  | Sensitivity analysis (9917) | Depression 2001-1990 | 0.17 | 0.04 | 0.08, 0.25 | 3.81 | .002** |
|  |  | Depression 2011-2001 | -0.20 | 0.04 | -0.29, -0.11 | -4.60 | <.001*** |
|  |  | Depression 2020-2011 | -0.05 | 0.05 | -0.15, 0.04 | -1.14 | .381 |
|  |  | Schizophrenia 2001-1990 | 0.10 | 0.04 | 0.01, 0.19 | 2.27 | .068 |
|  |  | Schizophrenia 2011-2001 | -0.09 | 0.05 | -0.18, 0.00 | -1.95 | .121 |
|  |  | Schizophrenia 2020-2011 | 0.05 | 0.05 | -0.04, 0.15 | 1.08 | .397 |
| Sympathy | Represen-tative West German population sample (10366) | Depression 2001-1990 | 0.23 | 0.05 | 0.14, 0.32 | 4.97 | <.001*** |
|  |  | Depression 2011-2001 | -0.08 | 0.05 | -0.17, 0.01 | -1.82 | .152 |
|  |  | Depression 2020-2011 | 0.04 | 0.05 | -0.06, 0.13 | 0.82 | .575 |
|  |  | Schizophrenia 2001-1990 | 0.08 | 0.04 | -0.01, 0.16 | 1.71 | .163 |
|  |  | Schizophrenia 2011-2001 | -0.11 | 0.05 | -0.20, -0.02 | -2.49 | .045* |
|  |  | Schizophrenia 2020-2011 | -0.04 | 0.05 | -0.13, 0.06 | -0.73 | .599 |
|  | Sensitivity analysis (9917) | Depression 2001-1990 | 0.23 | 0.05 | 0.14, 0.32 | 5.05 | <.001*** |
|  |  | Depression 2011-2001 | -0.09 | 0.05 | -0.18, 0.00 | -1.91 | .121 |
|  |  | Depression 2020-2011 | 0.00 | 0.05 | -0.10, 0.10 | -0.07 | .941 |
|  |  | Schizophrenia 2001-1990 | 0.08 | 0.04 | -0.01, 0.17 | 1.81 | .145 |
|  |  | Schizophrenia 2011-2001 | -0.12 | 0.05 | -0.21, -0.03 | -2.58 | .037* |
|  |  | Schizophrenia 2020-2011 | -0.10 | 0.05 | -0.20, 0.00 | -1.91 | .121 |
| Compassion | Represen-tative West German population sample (10373) | Depression 2001-1990 | 0.15 | 0.04 | 0.07, 0.23 | 3.51 | .004** |
|  |  | Depression 2011-2001 | 0.02 | 0.04 | -0.06, 0.10 | 0.40 | .738 |
|  |  | Depression 2020-2011 | -0.05 | 0.04 | -0.14, 0.04 | -1.07 | .439 |
|  |  | Schizophrenia 2001-1990 | 0.12 | 0.04 | 0.03, 0.20 | 2.63 | .035* |
|  |  | Schizophrenia 2011-2001 | 0.15 | 0.04 | 0.06, 0.23 | 3.49 | .004** |
|  |  | Schizophrenia 2020-2011 | -0.09 | 0.04 | -0.18, -0.00 | -2.00 | .109 |
|  | Sensitivity analysis (9924) | Depression 2001-1990 | 0.15 | 0.04 | 0.07, 0.24 | 3.55 | .004** |
|  |  | Depression 2011-2001 | 0.01 | 0.04 | -0.07, 0.10 | 0.34 | .758 |
|  |  | Depression 2020-2011 | -0.05 | 0.05 | -0.14, 0.04 | -1.06 | .397 |
|  |  | Schizophrenia 2001-1990 | 0.12 | 0.04 | 0.03, 0.21 | 2.67 | .030* |
|  |  | Schizophrenia 2011-2001 | 0.15 | 0.04 | 0.06, 0.23 | 3.42 | .005** |
|  |  | Schizophrenia 2020-2011 | -0.11 | 0.05 | -0.20, -0.01 | -2.26 | .068 |
| Desire to help | Represen-tative West German population sample 10372 () | Depression 2001-1990 | 0.14 | 0.04 | 0.05, 0.23 | 3.20 | .008** |
|  |  | Depression 2011-2001 | -0.03 | 0.04 | -0.12, 0.05 | -0.73 | .599 |
|  |  | Depression 2020-2011 | 0.00 | 0.05 | -0.09, 0.09 | 0.02 | .980 |
|  |  | Schizophrenia 2001-1990 | -0.15 | 0.04 | -0.23, -0.07 | -3.49 | .004** |
|  |  | Schizophrenia 2011-2001 | -0.08 | 0.05 | -0.17, 0.01 | -1.74 | .157 |
|  |  | Schizophrenia 2020-2011 | 0.03 | 0.05 | -0.06, 0.12 | 0.67 | .615 |
|  | Sensitivity analysis (9920) | Depression 2001-1990 | 0.14 | 0.04 | 0.05, 0.23 | 3.20 | .008** |
|  |  | Depression 2011-2001 | -0.03 | 0.04 | -0.12, 0.05 | -0.76 | .539 |
|  |  | Depression 2020-2011 | -0.05 | 0.05 | -0.15, 0.04 | -1.07 | .397 |
|  |  | Schizophrenia 2001-1990 | -0.15 | 0.04 | -0.23, -0.06 | -3.46 | .005** |
|  |  | Schizophrenia 2011-2001 | -0.08 | 0.05 | -0.17, 0.01 | -1.77 | .153 |
|  |  | Schizophrenia 2020-2011 | -0.06 | 0.05 | -0.16, 0.04 | -1.19 | .361 |
| Lack of understanding | Represen-tative West German population sample (10372) | Depression 2001-1990 | 0.07 | 0.05 | -0.02, 0.16 | 1.48 | .231 |
|  |  | Depression 2011-2001 | -0.06 | 0.05 | -0.15, 0.03 | -1.37 | .278 |
|  |  | Depression 2020-2011 | -0.19 | 0.05 | -0.28, -0.09 | -3.94 | .001** |
|  |  | Schizophrenia 2001-1990 | 0.05 | 0.05 | -0.05, 0.15 | 0.95 | .488 |
|  |  | Schizophrenia 2011-2001 | -0.01 | 0.05 | -0.11, 0.09 | -0.24 | .840 |
|  |  | Schizophrenia 2020-2011 | -0.09 | 0.05 | -0.20, 0.01 | -1.78 | .155 |
|  | Sensitivity analysis (9921) | Depression 2001-1990 | 0.07 | 0.05 | -0.02, 0.17 | 1.57 | .209 |
|  |  | Depression 2011-2001 | -0.07 | 0.05 | -0.16, 0.02 | -1.45 | .244 |
|  |  | Depression 2020-2011 | -0.13 | 0.05 | -0.23, -0.03 | -2.56 | .037* |
|  |  | Schizophrenia 2001-1990 | 0.05 | 0.05 | -0.05, 0.15 | 1.04 | .397 |
|  |  | Schizophrenia 2011-2001 | -0.02 | 0.05 | -0.11, 0.08 | -0.32 | .758 |
|  |  | Schizophrenia 2020-2011 | -0.03 | 0.06 | -0.13, 0.08 | -0.45 | .709 |

Contrasts of multiple regression analyses with emotional reactions as criterion and period, vignette, interaction of period*vignette, age, and gender of vignette or respondents as predictors. The contrast estimates describe the change per period and vignette. Positive values indicate increased emotional reactions. The sensitivity analyses exclude self-administered interviews, an option specific to 2020 due to the COVID-19 pandemic. The *p* values are corrected for separate testing of items, periods, and vignettes using the Benjamini-Hochberg procedure to control the false discovery rate at *q* = .05. * *p*<.05, ** *p*<.01, *** *p*<.001.
